# Ocular and visual perceptive factors associated with treatment outcomes in patients with anisometropic amblyopia

**DOI:** 10.1186/s12886-023-02770-2

**Published:** 2023-01-12

**Authors:** Jie Hong, Debbie Kuo, Han Su, Lei Li, Yanan Guo, Hang Chu, Jing Fu

**Affiliations:** 1 Beijing Ophthalmology & Visual Sciences Key Lab, Dongcheng District, Beijing Tongren Eye Center, Beijing Tongren Hospital, Capital Medical University, 1 Dongjiaominxiang Street, Beijing, China; 2 Palo Alto Medical Foundation, Palo Alto, CA USA; 3 Guangdong Medical Device Research Institute, Guangzhou, China

**Keywords:** Risk factors, Anisometropic amblyopia, Suppression, Perceptual eye position (PEP), Stereoacuity

## Abstract

**Background:**

The aim of this observational study was to identify ocular and visual perceptive risk factors related to treatment results following refractive correction and patching in children with anisometropic amblyopia, who were between the ages of 4 to 14 years old.

**Methods:**

One-hundred and two children with newly diagnosed anisometropic amblyopia were recruited. Successful treatment of amblyopia was defined as the final best corrected visual acuity (BCVA) better than or equal to 0.1 logMAR and amblyopic eye BCVA within 1 line of the sound eye BCVA by the end of the treatment period. BCVA, cycloplegic refraction, stereoacuity, perceptual eye position (PEP) and interocular suppression were measured.

**Results:**

Of these patients, 45.10% achieved successful treatment of amblyopia after refractive correction and patching for 10.5 months. The mean age was not significantly different between patients who were successfully and unsuccessfully treated (5.50 ± 1.59 years vs 6.14 ± 2.19 years, respectively). Patients who failed treatment had significantly larger interocular difference of BCVA at the time of initial treatment (successful group: 0.33 ± 0.29 logMAR, unsuccessful group: 0.65 ± 0.35 logMAR) and after refractive adaptation (successful group: 0.15 ± 0.13 logMAR, unsuccessful group: 0.42 ± 0.35 logMAR). They also had higher spherical equivalent (SE) of amblyopic eyes (successful group: 3.08 ± 3.61 D, unsuccessful group: 5.27 ± 3.38 D), bigger interocular difference of SE (successful group: 0.94 ± 2.71 D, unsuccessful group: 3.09 ± 3.05 D), worse stereoacuity (successful group: 2.32 ± 0.37 log seconds of arc, unsuccessful group: 2.75 ± 0.32 log seconds of arc), larger vertical PEP deviation (successful group: 6.41 ± 6.08 pixel, unsuccessful group: 19.07 ± 24.96 pixel) and deeper interocular suppression (successful group: 21.7 ± 19.7%, unsuccessful group: 37.8 ± 27.1%) than those of successfully treated patients. The most influential treatment failure risk factors were larger vertical PEP deviation [adjusted odds ratio (OR) (95% confidence interval) 1.12 (1.02–1.22)] and worse stereoacuity [adjusted odds ratio (OR) (95% confidence interval) 7.72 (1.50–39.85)] in multiple logistic regression analysis.

**Conclusions:**

Larger vertical PEP deviation and worse stereoacuity were the most influential treatment failure risk factors in children with anisometropic amblyopia. The vertical PEP deviation and stereoacuity, which can reflect interocular interaction, may be useful in predicting the response to therapy.

## Background

Amblyopia is believed to be caused by an abnormal visual experience that occurs during the period of early childhood or infancy [1]. Anisometropic amblyopia is a common type of amblyopia. Several researchers have found that factors such as high spherical equivalent (SE) [2], age over six years old, the presence of astigmatism, and poor initial best-corrected visual acuity (BCVA) may affect the treatment outcome for anisometropia amblyopia [3, 4].

Amblyopia is also considered to be a neurodevelopmental disease in which the visual cortex receives discordant input from each eye. It is believed that amblyopia is not only characterized by reduced BCVA but also by deficits of the cortex. Recently, interocular interaction, including factors of suppression and fixation disparity, has received increasing attention from researchers [5, 6]. It has been reported that suppression varies among different types of amblyopia [7] and is associated with the severity of amblyopia. While interocular suppression plays a primary role in amblyopia and has implications for the treatment of amblyopia [8], the relationship between visual acuity improvement from amblyopic treatment and suppression is complex [7, 9].

Another area of investigation is oculomotor control. The oculomotor influences on visual processing, which is thought to serve the function of perceptual stability, play an essential role in visual plasticity [10]. A growing body of evidence supports that amblyopia impairs some aspects of oculomotor control [11, 12] and some researchers have found decreased fixation stability in the amblyopic eye [6, 13, 14]. The perceptual eye position (PEP) test, developed by H.J Haasel, can measure simultaneously vertical and horizontal deviation which reflect fixation disparity [15]. It has been reported that the degree of vertical PEP deviation is related with the severity of anisometropia, with bigger interocular SE differences associated with higher deviation of vertical PEP [16]. Visual information seen by a suppressed amblyopic eye can be binocularly integrated and influence the overall visual perception [17]. The interocular interaction may reflect abnormalities in the visual cortex, but it is currently unknown if visual perceptual factors are associated with the results of amblyopia treatment. The aim of this study was to investigate ocular and visual perceptive factors that may impact the treatment outcomes in patients with anisometropic amblyopia.

### Patients and methods

#### Patients

This is an observational study of 102 children with anisometropic amblyopia from the Department of Ophthalmology at Beijing Tongren Hospital of Capital Medical University (Beijing, China) between January 1, 2020 and December 31, 2021. The inclusion criteria were as follows: (1) age 4 to 14 years old; (2) ability to complete examinations for BCVA, stereopsis, perceptual eye position (PEP) and suppression; (3) BCVA differing by at least two lines between eyes; (4) interocular difference in SE (spherical equivalent) of at least 1 diopter (D); (5) absence of structural ocular abnormalities in either eye and absence of strabismus; (6) children with new diagnosis of anisometropic amblyopia who had never been treated for the condition.

This study was performed in accordance with the Declaration of Helsinki and was approved by the ethics committee of the Beijing Tongren Hospital Institutional Review Board at Capital Medical University (TRECKY2018-024). All participants involved were informed of the purpose of this study. A written informed consent was obtained from their parents or legal guardians.

#### Eye examinations

Patients underwent all the initial ophthalmologic measurements as follows. Full cycloplegia was obtained after instillation of topical 1% atropine, twice a day for 3 days prior to their visit. Objective refraction with an autorefractor (Topcon KR8900, Tokyo, Japan) and retinoscopy were obtained before subjective refraction. BCVA was examined in both amblyopic and fellow eyes following cycloplegic refraction using a logMAR chart. The anterior eye segment was checked by slit lamp (Haag-Streit AG, Switzerland). Fundus photography was performed by using digital camera (Cannon CR-2, Japan). IOP was measured by non-contact tonometer (NCT) (TX-F, Canon, Japan). To rule out strabismus and abnormalities of extraocular muscles (EOM), cover testing and ocular motility examinations were performed. All patients were followed up at 4.5, 7.5 and 10.5 months after refractive correction and patching were initiated. To rule out the effects of refractive error itself on BCVA [18], stereoacuity, suppression and PEP, measurements of these data from the 4.5-month treatment follow up were used for statistical analysis. This timepoint was chosen to allow adequate time for full spectacle adaptation. At each follow up visit, repeat measurements of BCVA were taken. An evaluation system designed by the Guangdong Medical Device Research Institute was used to measure PEP, interocular suppression and stereoacuity, and the details of the testing protocols are below. The stimulating template was generated by MATLAB.

#### Assessment of stereoacuity

Identification of the E shape was used to assess the stereoacuity. The Randot test was tested at disparities of 400, 300, 200, and 100 s of arc. Nil stereo was recorded for patients who could not identify the E shapes at the 400 s of arc level. The stereoacuity results of seconds of arc were converted to logarithmic form for statistical analyses [19]. Nil stereo was assigned as 2.9 log seconds of arc for analyses (Table 1).

**Table 1:** Levels of stereoacuity showing equivalent Log seconds of Arc values

Seconds of Arc	Log of seconds of Arc
100	2.00
200	2.30
300	2.48
400	2.60
Nil stereo	2.90

#### Examination of perceptual eye position (PEP)

**Fig. 1:**
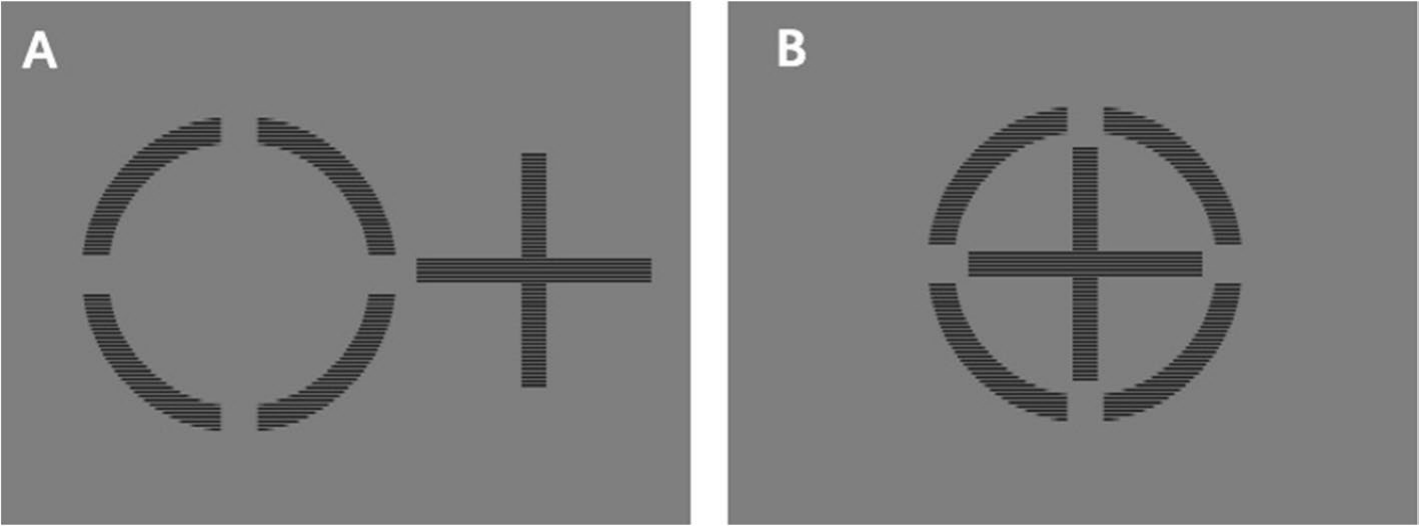
Measurement of PEP. Patients performed task while wearing polarized glasses, which allowed right eye to see a circle and left eye to see a cross (**A**). They were instructed to place the cross into the circle’s center by using a computer mouse (**B**). The horizontal and vertical deviation were automatically recorded by the system

The PEP tests were conducted according to the method reported previously [16]. Briefly, patients performed task while wearing polarized glasses, which allowed right eye to see a circle and left eye to see a cross. Patients were instructed to place the cross into the circle’s center by using a computer mouse. The stimulating template was as follows: the circle was 0.4 × 0.4° and the cross was 0.33 × 0.33°. The minimum unit of ocular misalignment observed by this computer-controlled ocular misalignment system was 1 pixel [20]. The horizontal and vertical deviation measured in pixels were automatically recorded by the system (shown in Fig. 1A and 1B).

#### Suppression measurement

**Fig. 2:**
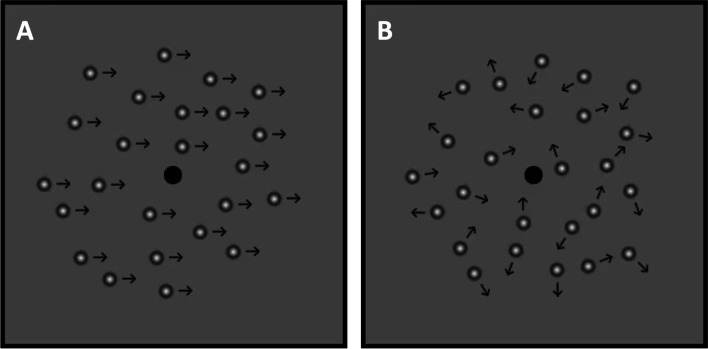
Measurement of suppression. The stimuli consist of two populations of moving dots. The 'signal' dots: moving in a common direction (**A**) and the 'noise' dots: moving in random directions (**B**). The patients performed task while wearing polarized glasses, which allowed one eye to see signal dots and the fellow eye to see noise dots. The task was to identify the motion direction of the signal dots

Dichoptic motion coherence test (National Engineering Research Center for Healthcare Devices, China). The method for evaluating interocular suppression has been described in detail previously [5, 8, 21]. Briefly, the stimuli consist of two populations of moving dots, one population moving in random directions (the 'noise' dots) and the other moving in a common direction (the 'signal' dots) (shown in Fig. 2A and 2B). Stimuli were generated using a computer (Windows XP system, PC host). Stimuli were presented on polarized 3 dimensional (3D) monitor (LG2342p, Korea) at a distance of 80 cm from the participant eyes. The task was to identify the motion direction of the signal dots. The first step of the test was to assess motion coherence threshold, that is the threshold number of signal dots required when both signal and noise dots are presented to both eyes at high contrast simultaneously. The second step was to measure the contrast imbalance between the eyes required to achieve the same threshold. Participants wore 3D polarized glasses to see a separate image for each eye, which allowed one eye to see signal dots and the fellow eye to see noise dots. The 3-down, 1-up staircase method was used for the psychophysical measurements in this procedure, and each staircase was repeated at least 3 times. According to the staircase algorithm, identification the motion direction of the signal dots resulted in an increase in the contrast of the noise dots shown to the fellow eye. During the examination, the contrast of the noise dots was varied by the staircase until performance level converged on 80% correct. This indicated that the noise and signal dots were being combined between the two eyes to produce the same level of task performance that was determined in the first step [21].

The signal was presented to the amblyopic eye and the noise was presented to the fellow eye and compared with vice versa. Firstly, signal dots fixed at high level (100%) contrast was presented to the amblyopic eye and noise dots were presented to the fellow eye. The contrast of noise dots was gradually increased at 10 levels (10%, 20%, 30%, 40%, 50%, 60%, 70%, 80%, 90% and 100% contrast) until threshold task performance occurred. Secondly, signal dots were presented to the fellow eye at a fixed high contrast and gradually increasing contrast of noise dots were presented to the amblyopic eye until threshold task performance occurred. The interocular difference in the contrast at threshold reflects the degree of interocular suppression with bigger interocular difference in the contrast indicating stronger suppression.

#### Therapeutic methods

Spectacles were prescribed based on retinoscopy after cycloplegia. Anisometropia, astigmatism, and myopia were fully corrected. Hyperopia was either fully corrected or symmetrically under-corrected by no more than + 2.0 D in both eyes. All the patients were prescribed with spectacles alone for 4.5 months followed by a phase of occlusion. The dosage of occlusion was prescribed according the severity of amblyopia and interocular differences in BCVA, generally following the guidance of PEDIG studies with some minor adjustments [22, 23]. For severe amblyopia (BCVA in the amblyopic eye ≥ 0.7 logMAR), patients were instructed to patch for 6 h/d. For moderate amblyopia (BCVA in the amblyopic eye 0.3–0.6 logMAR), the patching dose was 2 h/d if the interocular difference was less than 4 lines and 4 h/d if the interocular difference was equal to or more than 4 lines. For mild amblyopia (BCVA in amblyopic eye ≤ 0.2 logMAR), patients were prescribed patching for 2 h/d. The patching dosage was adjusted at each follow up. Patient compliance was supervised by their parents and assessed by a calendar on which parents recorded the completion of the treatment each day. The calendars were reviewed by the investigator at follow-up visits.

#### Criteria for grouping by treatment results

In this study, patients were separated into two subgroups according to the results of treatment after being treated for 10.5 months: successful group (patients with resolution of amblyopia) and unsuccessful group (patients who did not achieve resolution of amblyopia). Resolution of amblyopia was defined as the final BCVA being better than or equal to 0.1 logMAR and amblyopic eye BCVA within 1 line of fellow eye BCVA by the end of the treatment period [24].

For univariate and multiple logistic regression analysis, the patients were divided into groups based on the spherical equivalent (SE) of the amblyopic eye (≤ 3D vs. > 3D), the interocular difference in SE (≤ 3D vs. > 3D), and deeper interocular suppression (the interocular difference in the contrast at threshold larger than 40% vs. ≤ 40%).

#### Statistical analysis

SPSS Statistics 22.0 (IBM) was used for statistical analyses. Descriptive statistics were used to present characteristics of the study groups. The comparisons of BCVA, SE, the interocular differences of BCVA and SE, stereoacuity, PEP, and interocular suppression between successful group and unsuccessful group were made using the independent sample T-test. Changes in BCVA, stereoacuity, PEP, interocular suppression after treatment between groups were evaluated using a paired T-test. Univariate and multiple logistic regression analyses were used to identify the risk factors for treatment outcomes of anisometropic amblyopia. Missing data were handled through deletion. *P*-values less than 0.05 were considered to be statistically significant.

## Results

A total of 102 patients were enrolled in this study and 92 patients completed the treatment and follow up. The patients who did not complete the study were lost to follow-up and their data were not included in analysis.

At study entry, mean BCVA in the amblyopic eyes at study entry was 0.57 ± 0.36 logMAR and in the fellow eye was 0.06 ± 0.09 logMAR. The mean SE in the amblyopic eye was 4.29 ± 3.63 D. Overall, 45.10% achieved resolution of amblyopia after refractive correction and patching at 10.5 months.

### Age

**Fig. 3:**
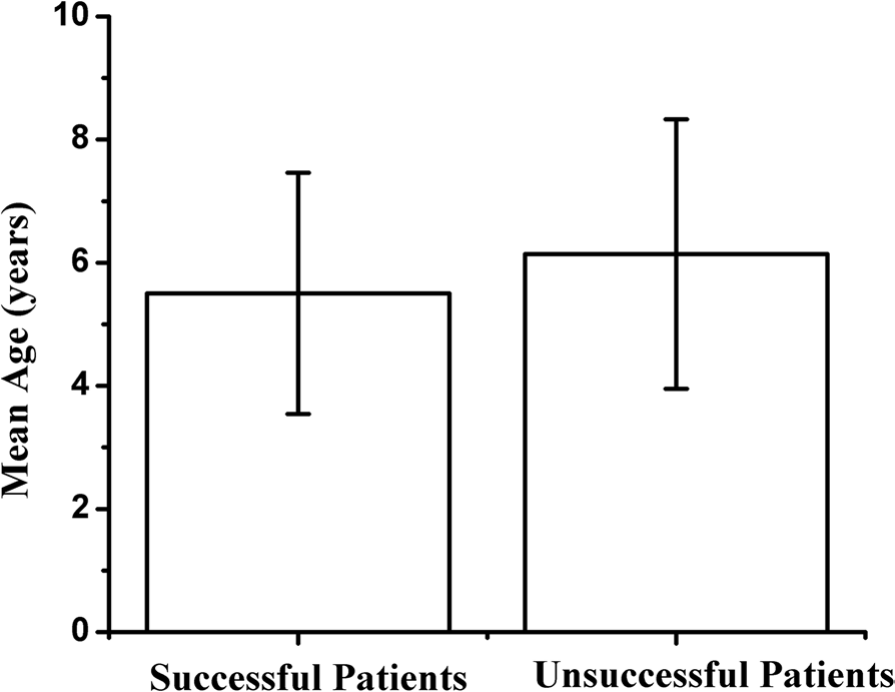
Comparison of age between successful and unsuccessful groups

The mean age was 5.85 ± 1.96 years (range: 4 to 14 years). Among these patients, 90.2% were ≤ 8 years of age. The mean age of patients in the successful and unsuccessful groups were 5.50 ± 1.59 years and 6.14 ± 2.19 years, respectively. The difference in mean age between two groups was not significant (*P* = 0.10) (Fig. 3) (Table 2). Age was not a risk factor both in univariate and multiple logistic regression analysis (*P* = 0.11, *P* = 0.14) (Table 4).

**Table 2:** The comparison of characteristics between the successful and unsuccessful groups

Characteristic	Successful group	Unsuccessful group	*P* value
Age (years)	5.50 ± 1.59	6.14 ± 2.19	0.10
Initial BCVA in amblyopic eyes (logMAR units)	0.40 ± 0.28	0.71 ± 0.35	< 0.01
Initial interocular difference in BCVA (logMAR units)	0.33 ± 0.29	0.65 ± 0.35	< 0.01
BCVA in amblyopic eyes after refractive adaptation (logMAR units)	0.18 ± 0.13	0.45 ± 0.33	< 0.01
Interocular difference of BCVA after refractive adaptation (logMAR units)	0.15 ± 0.13	0.42 ± 0.35	< 0.01
SE in amblyopic eyes (D)	3.08 ± 3.61	5.27 ± 3.38	< 0.01
Interocular SE refraction difference (D)	0.94 ± 2.71	3.09 ± 3.05	< 0.01
Stereoacuity (Log seconds of Arc)	2.32 ± 0.37	2.75 ± 0.32	< 0.001
Horizontal PEP deviation (pixel)	39.41 ± 43.53	45.36 ± 62.71	0.60
Vertical PEP deviation (pixel)	6.41 ± 6.08	19.07 ± 24.96	< 0.01
Interocular difference in contrast	21.7 ± 19.7%	37.8 ± 27.1%	< 0.01

Results are given as the mean ± SD (standard deviation)

*BCVA* Best-corrected visual acuity, *logMAR* Logarithm of the minimum angle of resolution, *SE* Spherical equivalent, *D* Diopters, *PEP* Perceptual eye position

### BCVA

**Fig. 4:**
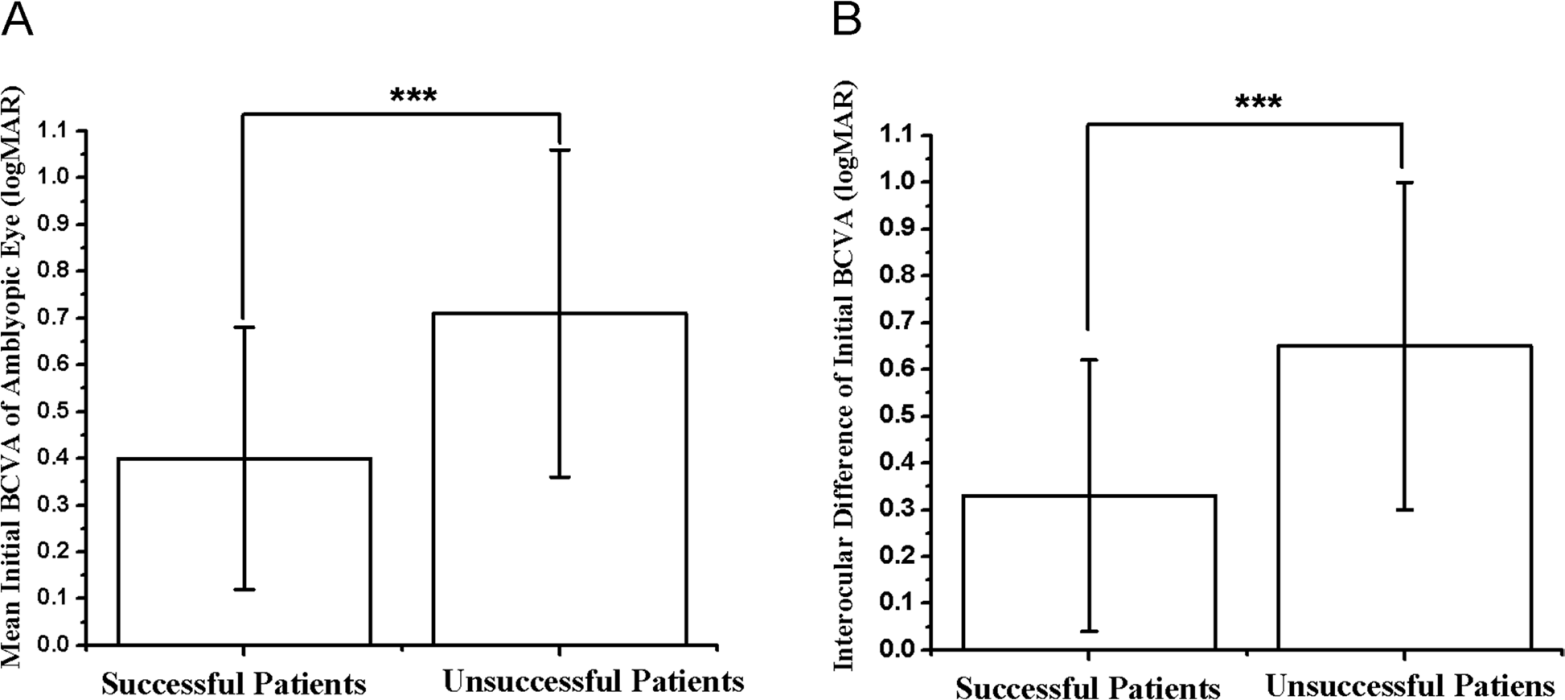
Comparison of initial BCVA of amblyopic eye (**A**), interocular difference of initial BCVA (**B**) between successful and unsuccessful groups, *** < 0.01

**Fig. 5:**
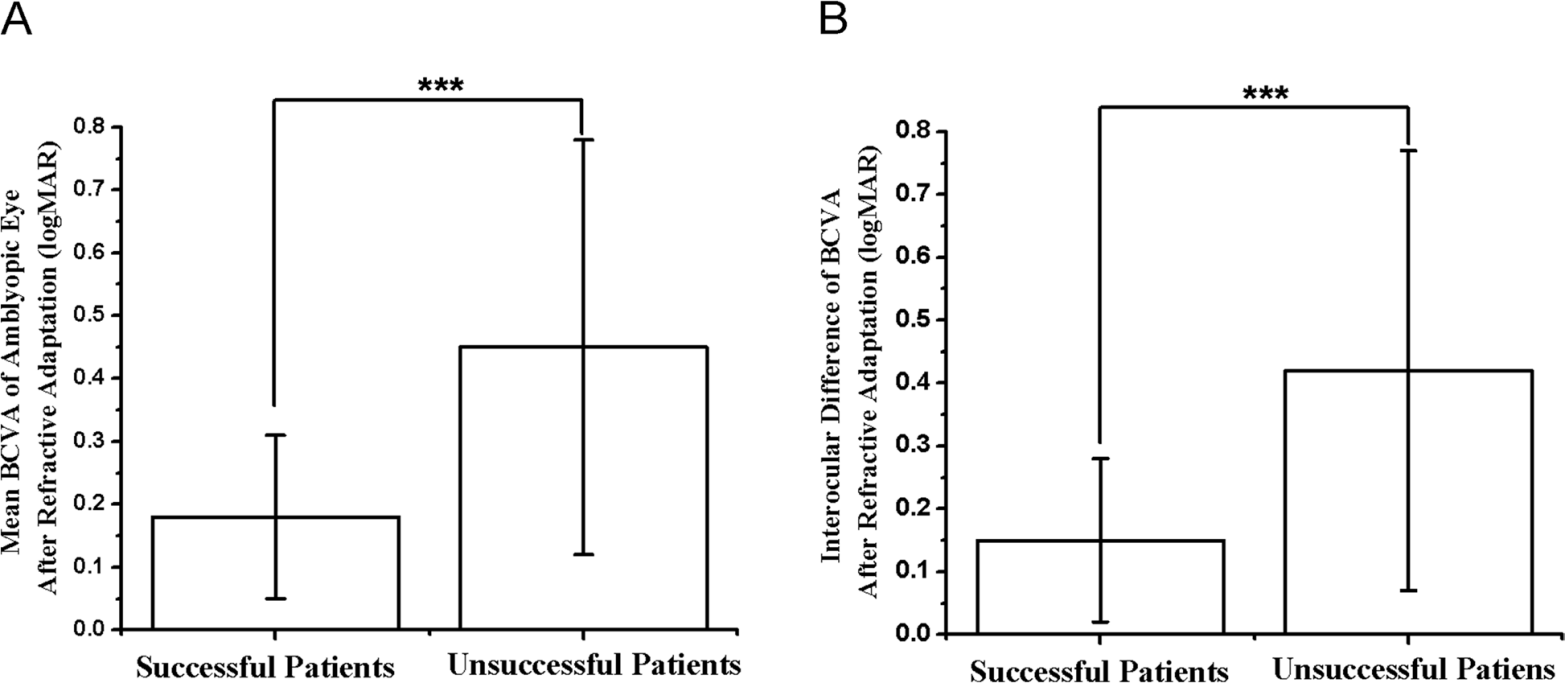
Comparison of BCVA of amblyopic eye (**A**), interocular difference of initial BCVA (**B**) between successful and unsuccessful groups after refractive adaptation, *** < 0.01

The mean initial BCVA of amblyopic eyes in the successful group and the unsuccessful group were 0.40 ± 0.28 logMAR units and 0.71 ± 0.35 logMAR units, respectively. Patients in the successful group had better BCVA than those in the unsuccessful group (t = -4.83, *P* < 0.01) (Fig. 4A). The mean interocular difference in initial BCVA in the successful group and the unsuccessful group were 0.33 ± 0.29 logMAR units and 0.65 ± 0.35 logMAR units, respectively (Fig. 4B). After refractive adaptation for 4.5 month, the mean BCVA of amblyopic eyes in the successful group and the unsuccessful group were 0.18 ± 0.13 logMAR units and 0.45 ± 0.33 logMAR units, respectively. Patients in the successful group had better BCVA than those in the unsuccessful group at the 4.5-month visit (*P* < 0.01) (Fig. 5A). The mean interocular difference in BCVA in the successful group and the unsuccessful group were 0.15 ± 0.13 logMAR units and 0.42 ± 0.35 logMAR units, respectively (Fig. 5B) (Table 2). After treatment for 10.5 months, the mean BCVA of amblyopic eyes in the successful group and the unsuccessful group was 0.03 ± 0.04 logMAR units and 0.20 ± 0.17 logMAR units, respectively. The BCVA of amblyopic eyes was significantly improved after treatment in both groups (*P* < 0.01, *P* < 0.01) (Table 3). Univariate analysis showed bigger interocular difference of BCVA both before and after refractive adaptation were significantly correlated with therapy outcome (*P* < 0.01, *P* < 0.01). However, they were not independent risk factors in multiple logistic regression analysis (*P* = 0.91, *P* = 0.09) (Table 4).

**Table 3:** Change in characteristics after treatment between the successful and unsuccessful groups

Characteristic	Successful Group		*P* value	Unsuccessful group		*P* value
	After refractive adaption	Final follow-up		After refractive adaption	Final follow-up	
BCVA in amblyopic eye (logMAR units)	0.18 ± 0.13	0.03 ± 0.04	< 0.01	0.45 ± 0.33	0.20 ± 0.17	< 0.01
Stereoacuity (Log seconds of Arc)	2.32 ± 0.37	2.17 ± 0.27	0.04	2.75 ± 0.32	2.55 ± 0.36	< 0.01
Horizontal PEP deviation (pixel)	39.41 ± 43.53	38.04 ± 44.72	0.95	45.36 ± 62.71	45.19 ± 65.50	0.51
Vertical PEP deviation (pixel)	6.41 ± 6.08	8.13 ± 9.14	0.31	19.07 ± 24.96	16.00 ± 29.23	0.37
Interocular difference in contrast	21.7 ± 19.7%	18.0 ± 15.9%	0.29	37.8 ± 27.1%	31.1 ± 22.5%	0.15

Results are given as the mean ± SD (standard deviation)

*BCVA* Best-corrected visual acuity, *logMAR* Logarithm of the minimum angle of resolution, *PEP* Perceptual eye position

**Table 4:** Multiple logistic regression analysis of suspected risk factors affecting treatment outcomes

Factor	Unadjusted OR (95% CI)	P	Adjusted OR (95% CI)	P
Age	1.20 (0.96–1.49)	0.11	1.31 (0.92–1.88)	0.14
SE refraction of amblyopic eye > 3D	4.57 (1.82–11.50)	< 0.01	2.75 (0.60–12.48)	0.19
Interocular difference of SE > 3D	4.34 (1.80–10.45)	< 0.01	0.20 (0.03–1.39)	0.10
Interocular difference of initial BCVA	27.59 (5.34–142.44)	< 0.01	0.84 (0.04–16.24)	0.91
Interocular difference of BCVA after refractive adaptation	251.15 (16.68–4974.08)	< 0.01	93.32 (0.53–16,430.64)	0.09
Vertical PEP deviation	1.11 (1.05–1.18)	< 0.01	1.12 (1.02–1.22)	0.01
Stereoacuity	21.00 (5.81–75.92)	< 0.01	7.72 (1.50–39.85)	0.02
Interocular suppression	5.27 (1.75–15.86)	< 0.01	2.86 (0.52–15.66)	0.23

*BCVA* Best-corrected visual acuity, *logMAR* Logarithm of the minimum angle of resolution, *SE* Spherical equivalent, *D* Diopters, *PEP* Perceptual eye position, *OR* Odds ratio, *CI* Confidence interval

### SE

**Fig. 6:**
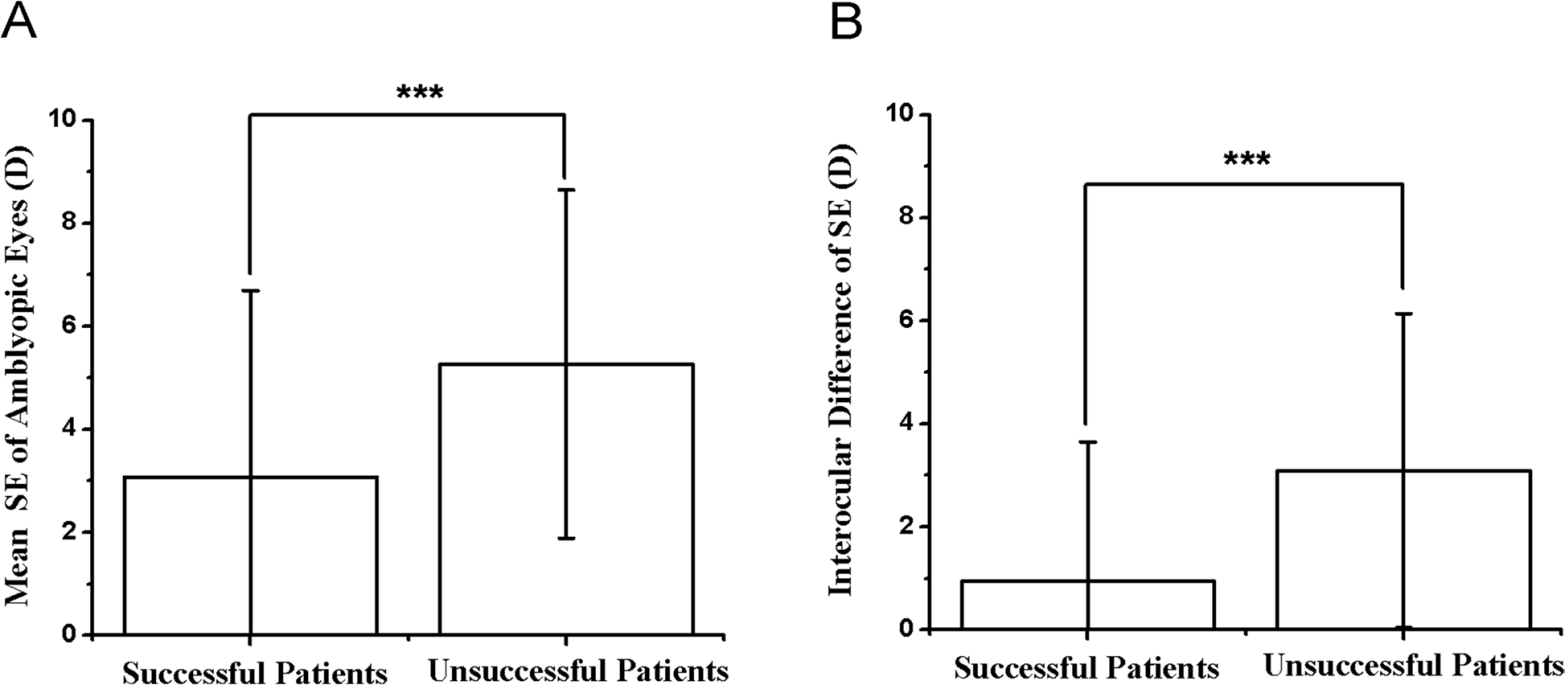
Comparison of SE of amblyopic eye (**A**), interocular difference of SE between successful and unsuccessful groups, *** < 0.01

The mean SE of amblyopic eyes in the successful and unsuccessful groups was 3.08 ± 3.61 D and 5.27 ± 3.38 D, respectively (Fig. 6A). The mean interocular difference of SE in the successful and unsuccessful groups was 0.94 ± 2.71 D and 3.09 ± 3.05 D, respectively (Fig. 6B). Both the mean SE of amblyopic eyes and the interocular difference of SE were significantly larger in patients without resolution of amblyopia compared to those with resolution (*P* < 0.01, *P* < 0.01) (Table 2). Although larger SE of amblyopic eyes and interocular difference of SE were found to be significantly correlated with treatment failure in univariate analysis (*P* < 0.01, *P* < 0.01), they were not independent risk factors after multiple logistic regression analysis (*P* = 0.19, *P* = 0.10) (Table 4).

### Stereoacuity

**Fig. 7:**
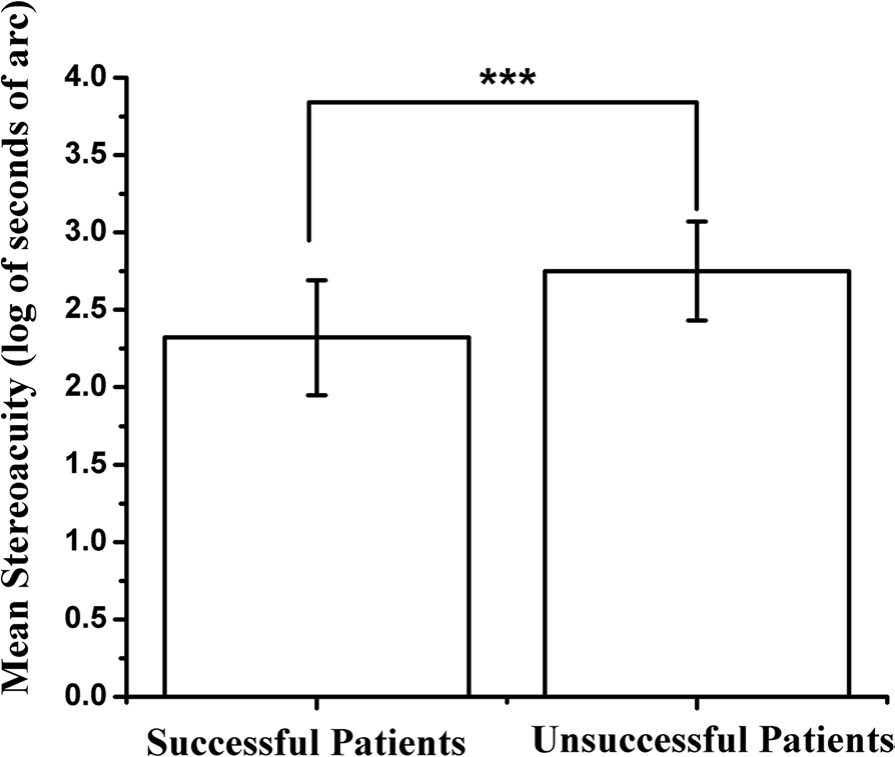
Comparison of stereoacuity after refractive adaptation between successful and unsuccessful groups, *** < 0.01

After 4.5 month of refractive adaptation, the stereoacuity of the successful and the unsuccessful group were 2.32 ± 0.37 and 2.75 ± 0.32 log seconds of arc, respectively. The successful group had significantly better stereoacuity than those of unsuccessful group (t = -5.87, *P* < 0.001) (Fig. 7) (Table 2). At the end of follow-up, the stereoacuity of the successful and the unsuccessful group were 2.17 ± 0.27 and 2.55 ± 0.36 log seconds of arc, respectively. Stereoacuity had a significant improvement both in successful and unsuccessful groups (*P* = 0.04, *P* < 0.01) (Table 3). Worse stereoacuity was identified as a risk factor for treatment failure both in univariate (*P* < 0.01) and in multivariate logistic analysis (*P* = 0.02) [adjusted odds ratio (OR) (95% confidence interval, CI) 7.72 (1.50–39.85)] (Table 4).

### The effect of PEP deviation on visual acuity improvement

**Fig. 8:**
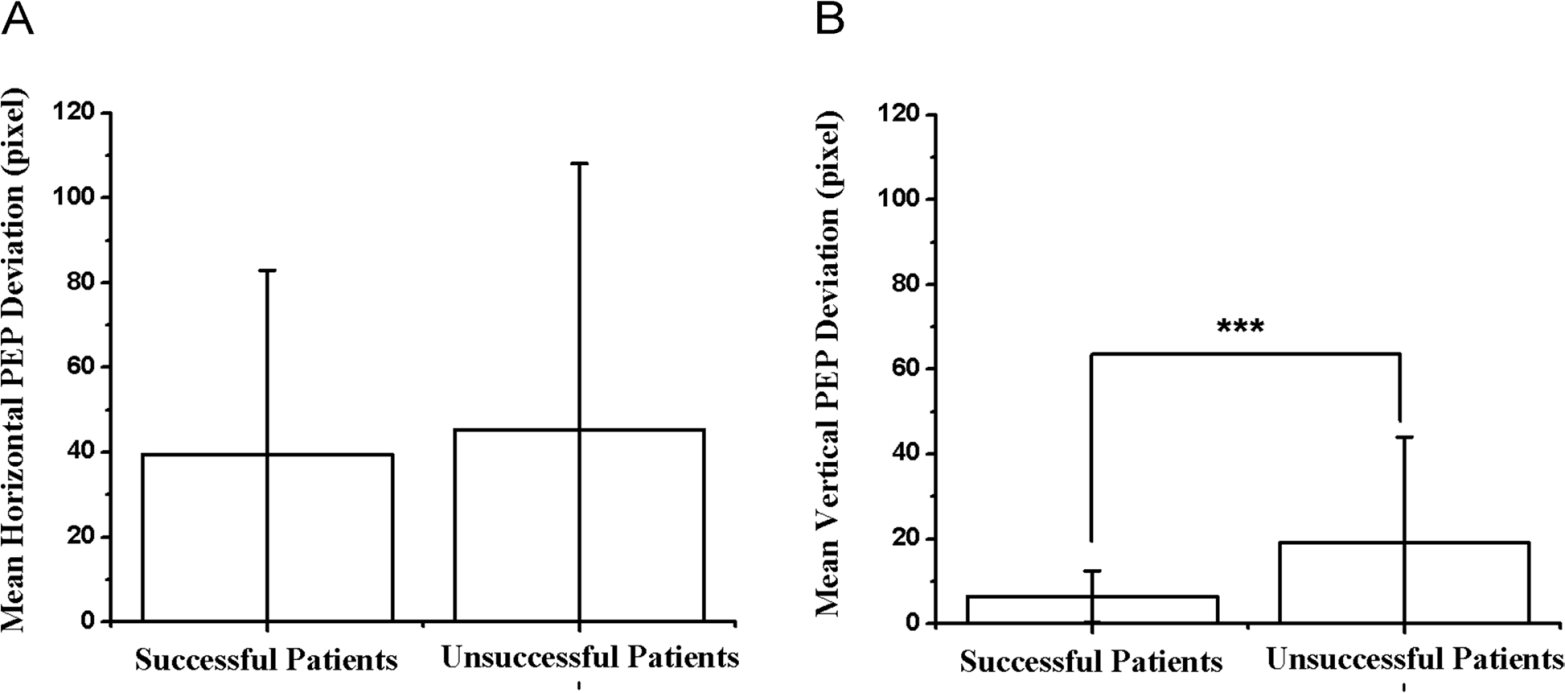
Comparison of horizontal PEP (**A**) and vertical PEP (**B**) after refractive adaptation between successful and unsuccessful groups, *** < 0.01

After 4.5 month of refractive adaptation, the mean horizontal PEP deviation of the successful and unsuccessful groups was 39.41 ± 43.53 and 45.36 ± 62.71 pixels, respectively. There was no significant difference in horizontal PEP between these two groups (t = -0.53, *P* = 0.60) (Fig. 8A). At the end of follow-up, the mean horizontal PEP deviation of the successful and unsuccessful groups was 38.04 ± 44.72 and 45.19 ± 65.50 pixels, respectively. The horizontal PEP did not show a significant change in either group (*P* = 0.95, *P* = 0.51) (Table 3). The mean vertical PEP deviation of the successful and unsuccessful groups was 6.41 ± 6.08 and 19.07 ± 24.96 pixel, respectively. The mean vertical PEP deviation in the unsuccessful group was significantly larger than those in the successful group (t = -3.34, *P* < 0.01) (Fig. 8B) (Table 2). At the end of follow-up, the mean vertical PEP deviation of the successful and unsuccessful groups was 8.13 ± 9.14 and 16.00 ± 29.23 pixels, respectively. There were no significant changes of vertical PEP deviation in either group (*P* = 0.31, *P* = 0.37) (Table 3). Larger vertical PEP deviation was a significant risk factor for treatment failure both in univariate (*P* < 0.01) and in multivariate analysis (*P* = 0.01) [adjusted OR (95% CI) 1.12 (1.02–1.22)] (Table 4).

### Interocular suppression and treatment outcomes

**Fig. 9:**
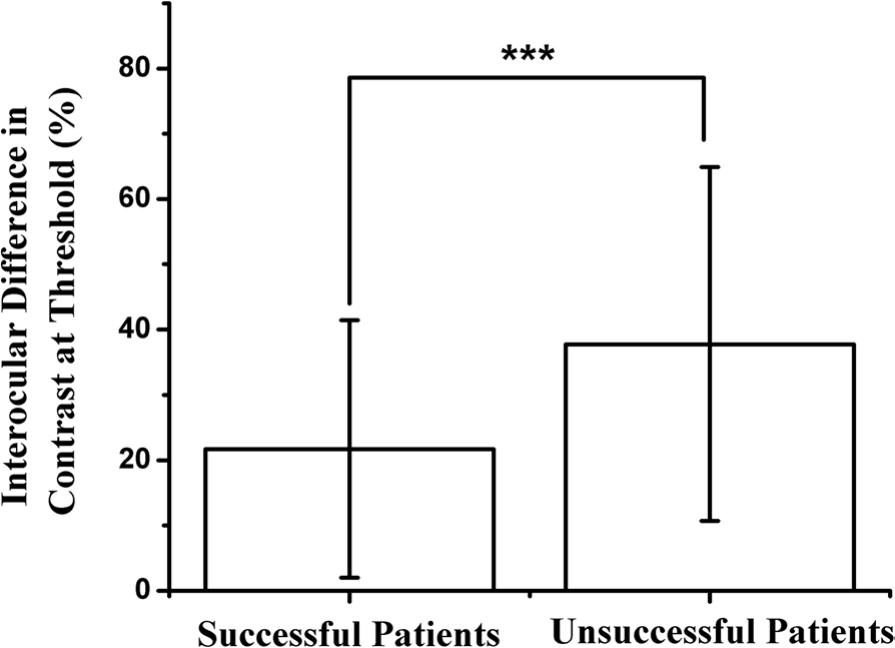
Comparison of interocular suppression after refractive adaptation between successful and unsuccessful groups, *** < 0.01

After 4.5 month of refractive adaptation, the interocular difference in the contrast at threshold in successful and unsuccessful groups was 21.7 ± 19.7% and 37.8 ± 27.1%, respectively. Patients with resolution of amblyopia had less suppression than those in the unsuccessful group (t = -3.26, *P* < 0.01) (Fig. 9) (Table 2). After 10.5-month therapy, the interocular difference in the contrast at threshold in successful and unsuccessful groups was 18.0 ± 15.9% and 31.1 ± 22.5%, respectively. Although the suppression decreased after treatment, the improvement was not statistically significant (*P* = 0.29, *P* = 0.15) (Table 3). In the univariate analysis, deeper suppression was significantly associated with the increased risk of treatment failure (*P* < 0.01). However, it was not identified as an independent risk factor in multiple logistic regression analysis (*P* = 0.23) (Table 4).

## Discussion

Amblyopia is a common cause of vision impairment in children and about a third of amblyopia is attributed to uncorrected anisometropia [25]. Amblyopia treatment success rates in the literature vary from 30.0% to 96.2% with different definition of success [4, 26, 27]. In this study, 45.10% of the patients achieved resolution of amblyopia. Timely identification of patients at high risk for treatment failure and prescribing targeted therapy provides the possibility for better prognosis.

Some studies have explored factors affecting outcomes of therapy for anisometropic amblyopia. However, the results on age, SE differences, and poor initial vision have been controversial. Hussein et al. reported a reduction in the treatment effects with increasing age [3]. Nevertheless, age was not reported an effect modifier in some large randomized treatment trials [23, 28]. Some differences in these trials could account for the different conclusions. Importantly, the patients’ age groups were different among those studies and the compliance to treatment regimens can be affected by age, with older children being more resistant to patching and less likely to be successful and achieve resolution of amblyopia. However, our results suggested age at treatment onset was not a predictive factor for final BCVA in the amblyopic eye. One of the possible reasons for this different conclusion might be the number of the older children was very small in our study, with only 10% children over 8 years of age.

Some studies have pointed to intraocular differences in SE being associated with final BCVA in children with anisometropic amblyopia [4], whereas others reported high SE as the most significant risk factor for therapeutic failure [2]. Our results demonstrated the high SE of amblyopic eyes and the bigger interocular difference of SE were not independent risk factors for therapeutic failure, even though they were significantly associated with poor treatment outcomes in univariate analysis. Similarly, Hussein et al.’s results showed neither the degree of SE nor the interocular difference in SE was an independent risk factor for therapeutic failure [3]. The reasons for these differences are not well recognized and further studies are needed to explore the specific reasons and mechanisms for these findings.

Previous studies also found vision of 20/200 or worse in the amblyopic eye was a risk factor for treatment failure [3]. Kirandi et al. reported patients with greater interocular difference in initial BCVA had the higher likelihood of treatment failure, though initial poor visual acuity did not necessarily imply a worse prognosis [2]. In our study, bigger differences in BCVA between the two eyes correlated with the higher failure rates. However, the interocular difference in BCVA was not found to be an independent risk factor for amblyopia resolution. The differences in the definition of treatment success might have contributed to these different results. Treatment failure was defined by the improvement of vision in some studies, while it was defined by the finial vision in other studies. For example, patients with very poor initial vision can have significant improvement but may not achieve a final normal vision. These patients might be classified as a treatment success in some studies and a treatment failure in others.

The results in our study suggested stereoacuity was a good prognostic indicator of treatment outcomes in patients with anisometropic amblyopia, which was consistent with the results published by Caputo et al. [29]. The relationship between vision and stereoacuity is complex in amblyopia. It is well known that decreased vision of one eye may result in reduced stereoacuity. Overall, worse vision seems to associate with worse stereoacuity. However, there is also some inter-individual variability. For example, some anisometropic amblyopia patients with decreased unilateral visual acuity still preserve excellent stereoacuity, but some patients rarely restored normal stereoacuity even if they had achieved normal visual acuity [30]. The inter-individual variance may reflect individual difference of binocular interaction in the brain. The dissociation between stereoacuity and visual acuity may support our results to some extent since stereoacuity was found to be an independent risk factor of therapeutic outcomes.

Recently, some novel methods were developed to measure visual perception which can reflect interocular interaction in amblyopia. In this study we use these approaches to evaluate PEP deviation and suppression in anisometropic amblyopia and analyzed the correlation between these factors and treatment results. Yang et al. reported the degree of vertical PEP deviation was related with the severity of anisometropia with bigger interocular SE differences associated with higher deviation of vertical PEP [16].

Our study is the first to analyze the association between PEP deviation and treatment outcomes in anisometropic amblyopia. We found vertical PEP deviation was an independent prognostic factor for treatment outcomes. Patients with larger vertical PEP deviation were more likely to have treatment failure.

Some researchers have studied binocular misalignment and fixation instability in hyperopic anisometropic children. The results suggested ocular motor development could be disrupted by the binocular decorrelation caused by anisometropia [31]. Previous studies have shown increased amplitude of fixational saccades were observed in amblyopic patients without nystagmus, which contributes to the instability in both the amblyopic eye and the fellow eye, and greater fixational saccade amplitude is associated with longer treatment duration [32, 33]. Several studies reported an association between fixation instability and stereoacuity deficits in amblyopic patients. Fixation instability increased with decreased stereoacuity [34, 35]. Patients with suboptimal part-time patching treatment response had greater fixational eye movements abnormalities [36]. Some researchers suggested fixation instability can serve as a biomarker in amblyopia which could be important in understanding the deficits in visual acuity and stereoacuity and predicting treatment effectiveness [37–39]. It is possible that treatment effectiveness could be predicted earlier, based on the detection of the larger vertical PEP deviation. Early detection paired with earlier perceptual learning and traditional treatment for these patients might speed up the time of recovery and lead to better treatment results.

Suppression plays an important role in amblyopia and has been associated with severity of amblyopia and stereopsis [8]. Our results showed patients with deeper suppression were more likely to have poor therapeutic effects, which was in line with recent studies [5], although in multiple logistic regression analysis it was not an independent risk factor. During cortical binocular combination, visual cortex processes the unbalanced weighting of inputs from each eye and suppresses the amblyopic eye. Some children with anisometropic amblyopia are unable achieve resolution of amblyopia even after strict adherence to refractive correction and occlusion therapy. Some studies showed children with amblyopia who reached their best visual acuity and failed to improve from additional patching achieved additional benefit in visual acuity after binocular game play [40]. Parts of suppression due to cortical mechanisms may not improve by spectacles and patching alone, resulting in residual amblyopia, and may benefit from other modalities of treatment. Measurement of the degree of suppression provides the possibility to predict the therapy effects and to intervene earlier with alternative treatments that may maximize outcomes.

There were several limitations of this study. One of the inclusion criteria was that the patients had the ability to complete the PEP examination, thus the conclusions are limited to those whose PEP could be evaluated. The sample size of this study was relatively small, making it difficult to determine the real effects of some factors, such as the types of refractive errors. An important limitation was that we did not objectively measure occlusion compliance. We included patients which were compliant based on parent report, which would bias our results toward lower rates of treatment success. Additional longitudinal studies on larger populations of children with anisometropic amblyopia may help to further elucidate the impact of ocular and visual perceptive factors on treatment outcomes.

Conclusions: The eyes with larger interocular difference of BCVA at the time of initial treatment and after refractive adaptation, higher SE of amblyopic eyes and bigger interocular difference of SE, worse stereoacuity, larger vertical PEP deviation and deeper interocular suppression were less likely to respond to treatment in children with anisometropic amblyopia. Notably, larger vertical PEP deviation and worse stereoacuity were the most influential treatment failure risk factors. The vertical PEP deviation and stereoacuity which can reflect interocular interaction, may be useful in predicting the response to therapy.

## Data Availability

The data are available from the corresponding author upon reasonable request.

## Abbreviations

BCVA: Best corrected visual acuity
SE: Spherical equivalent
PEP: Perceptual eye position

## References

[1] Chen AM, Cotter SA (2016). The Amblyopia Treatment Studies: Implications for Clinical Practice. Adv Ophthalmol Optom.

[2] Kirandi EU, Akar S, Gokyigit B, Onmez FEA, Oto S (2017). Risk factors for treatment failure and recurrence of anisometropic amblyopia. Int Ophthalmol.

[3] Hussein MA, Coats DK, Muthialu A, Cohen E, Paysse EA (2004). Risk factors for treatment failure of anisometropic amblyopia. J AAPOS.

[4] Woodruff G, Hiscox F, Thompson JR, Smith LK (1994). Factors affecting the outcome of children treated for amblyopia. Eye (Lond).

[5] Li J, Hess RF, Chan LY, Deng D, Yang X, Chen X, Yu M, Thompson B (2013). Quantitative measurement of interocular suppression in anisometropic amblyopia: a case-control study. Ophthalmology.

[6] González EG, Wong AM, Niechwiej-Szwedo E, Tarita-Nistor L, Steinbach MJ (2012). Eye position stability in amblyopia and in normal binocular vision. Invest Ophthalmol Vis Sci.

[7] Kehrein S, Kohnen T, Fronius M (2016). Dynamics of Interocular Suppression in Amblyopic Children during Electronically Monitored Occlusion Therapy: First Insight. Strabismus.

[8] Li J, Thompson B, Lam CS, Deng D, Chan LY, Maehara G, Woo GC, Yu M, Hess RF (2011). The role of suppression in amblyopia. Invest Ophthalmol Vis Sci.

[9] Chen Y, He Z, Mao Y, Chen H, Zhou J, Hess RF (2019). Patching and Suppression in Amblyopia: One Mechanism or Two?. Front Neurosci.

[10] Laamerad P, Guitton D, Pack CC (2020). Eye movements shape visual learning. Proc Natl Acad Sci U S A.

[11] Hou SW, Zhang Y, Christian L, Niechwiej-Szwedo E, Giaschi D (2022). Evaluating visuomotor coordination in children with amblyopia. Dev Psychobiol.

[12] Nouraeinejad A. The effect of amblyopia on saccadic eye movements. Int J Neurosci. 2022:1–2. Online ahead of print 10.1080/00207454.2022.2100775 35815630

[13] Wang S, Zou L, Tian T, Zhan A, Liu Y, Wen W, Liu H (2022). Fixation stability improvement after occlusion treatment for severe amblyopia. Int Ophthalmol.

[14] Wang S, Tian T, Zou L, Wu S, Liu Y, Wen W, Liu H (2021). Fixation Characteristics of Severe Amblyopia with Eccentric Fixation and Central Fixation Assessed by the MP-1 Microperimeter. Semin Ophthalmol.

[15] Kommerell G, Gerling J, Ball M, de Paz H, Bach M (2000). Heterophoria and fixation disparity: a review. Strabismus.

[16] Yang C, Li X, Zhang G, Lan J, Zhang Y, Chu H, Li J, Xie W, Wang S, Yan L (2017). Comparison of perceptual eye positions among patients with different degrees of anisometropia. Medicine (Baltimore).

[17] Chow A, Silva AE, Tsang K, Ng G, Ho C, Thompson B (2021). Binocular Integration of Perceptually Suppressed Visual Information in Amblyopia. Invest Ophthalmol Vis Sci.

[18] Holmes JM, Levi DM (2018). Treatment of amblyopia as a function of age. Vis Neurosci.

[19] Hatt SR, Mohney BG, Leske DA, Holmes JM (2008). Variability of stereoacuity in intermittent exotropia. Am J Ophthalmol.

[20] Tan F, Yang X, Chu H, Yan L, Wiederhold BK, Wiederhold M, Liao Y (2020). The Study of Perceptual Eye Position Examination and Visual Perceptual Training in Postoperative Intermittent Exotropes. Cyberpsychol Behav Soc Netw.

[21] Black JM, Hess RF, Cooperstock JR, To L, Thompson B (2012). The measurement and treatment of suppression in amblyopia. J Vis Exp.

[22] Repka MX, Beck RW, Holmes JM, Birch EE, Chandler DL, Cotter SA, Hertle RW, Kraker RT, Moke PS, Quinn GE (2003). A randomized trial of patching regimens for treatment of moderate amblyopia in children. Arch Ophthalmol.

[23] Holmes JM, Kraker RT, Beck RW, Birch EE, Cotter SA, Everett DF, Hertle RW, Quinn GE, Repka MX, Scheiman MM (2003). A randomized trial of prescribed patching regimens for treatment of severe amblyopia in children. Ophthalmology.

[24] Cotter SA, Foster NC, Holmes JM, Melia BM, Wallace DK, Repka MX, Tamkins SM, Kraker RT, Beck RW, Hoover DL (2012). Optical treatment of strabismic and combined strabismic-anisometropic amblyopia. Ophthalmology.

[25] Group PEDI (2002). The clinical profile of moderate amblyopia in children younger than 7 years. Arch Ophthalmol.

[26] Flynn JT, Cassady JC (1978). Current trends in amblyopia therapy. Ophthalmology.

[27] Lee SY, Isenberg SJ (2003). The relationship between stereopsis and visual acuity after occlusion therapy for amblyopia. Ophthalmology.

[28] Repka MX, Cotter SA, Beck RW, Kraker RT, Birch EE, Everett DF, Hertle RW, Holmes JM, Quinn GE, Sala NA (2004). A randomized trial of atropine regimens for treatment of moderate amblyopia in children. Ophthalmology.

[29] Caputo R, Frosini R, De Libero C, Campa L, Magro EF, Secci J (2007). Factors influencing severity of and recovery from anisometropic amblyopia. Strabismus.

[30] Levi DM, Knill DC, Bavelier D (2015). Stereopsis and amblyopia: A mini-review. Vision Res.

[31] Birch EE, Subramanian V, Weakley DR (2013). Fixation instability in anisometropic children with reduced stereopsis. J AAPOS.

[32] Chen D, Otero-Millan J, Kumar P, Shaikh AG, Ghasia FF (2018). Visual Search in Amblyopia: Abnormal Fixational Eye Movements and Suboptimal Sampling Strategies. Invest Ophthalmol Vis Sci.

[33] Scaramuzzi M, Murray J, Otero-Millan J, Nucci P, Shaikh AG, Ghasia FF (2019). Fixation instability in amblyopia: Oculomotor disease biomarkers predictive of treatment effectiveness. Prog Brain Res.

[34] Kelly KR, Cheng-Patel CS, Jost RM, Wang YZ, Birch EE (2019). Fixation instability during binocular viewing in anisometropic and strabismic children. Exp Eye Res.

[35] Murray J, Garg K, Ghasia F (2021). Monocular and Binocular Visual Function Deficits in Amblyopic Patients with and without Fusion Maldevelopment Nystagmus. Eye and brain.

[36] Scaramuzzi M, Murray J, Nucci P, Shaikh AG, Ghasia FF (2021). Fixational eye movements abnormalities and rate of visual acuity and stereoacuity improvement with part time patching. Sci Rep.

[37] Kang SL, Beylergil SB, Otero-Millan J, Shaikh AG, Ghasia FF. Fixational Eye Movement Waveforms in Amblyopia: Characteristics of Fast and Slow Eye Movements. J Eye Mov Res. 2019;12(6). 10.16910/jemr.12.6.9. 10.16910/jemr.12.6.9 PMC7962684 33828757

[38] Murray J, Gupta P, Dulaney C, Garg K, Shaikh AG, Ghasia FF (2022). Effect of Viewing Conditions on Fixation Eye Movements and Eye Alignment in Amblyopia. Invest Ophthalmol Vis Sci.

[39] Ghasia F, Wang J (2022). Amblyopia and fixation eye movements. J Neurol Sci.

[40] Li SL, Jost RM, Morale SE, Stager DR, Dao L, Stager D, Birch EE (2014). A binocular iPad treatment for amblyopic children. Eye (Lond).

